# *Who Is Who?* Interpretation of Multiple Occurrences of the Chinese Reflexive: Evidence from Real-Time Sentence Processing

**DOI:** 10.1371/journal.pone.0073226

**Published:** 2013-09-03

**Authors:** Lan Shuai, Tao Gong, Yicheng Wu

**Affiliations:** 1 Department of Electrical and Computer Engineering, Johns Hopkins University, Baltimore, Massachusetts, United States of America; 2 Department of Linguistics, University of Hong Kong, Hong Kong; 3 Center for the Study of Language and Cognition, Zhejiang University, Hangzhou, China; Stony Brook University, United States of America

## Abstract

Theoretical linguists claim that the notorious reflexive *ziji* ‘self’ in Mandarin Chinese, if occurring more than once in a single sentence, can take distinct antecedents. This study tackles possibly the most interesting puzzle in the linguistic literature, investigating how two occurrences of *ziji* in a single sentence are interpreted and whether or not there are mixed readings, i.e., these *ziji*s are interpretively bound by distinct antecedents. Using 15 Chinese sentences each having two *ziji*s, we conducted two sentence reading experiments based on a modified self-paced reading paradigm. The general interpretation patterns observed showed that the majority of participants associated both *ziji*s with the same local antecedent, which was consistent with Principle A of the Standard Binding Theory and previous experimental findings involving a single *ziji*. In addition, mixed readings also occurred, but did not pattern as claimed in the theoretical linguistic literature (i.e., one *ziji* is bound by a long-distance antecedent and the other by a local antecedent). Based on these results, we argue that: (i) mixed readings were due to manifold, interlocking and conflicting perspectives taken by the participants; and (ii) cases of multiple occurrences of *ziji* taking distinct antecedents are illicit in Chinese syntax, since the speaker, when expressing a sentence, can select only one P(erspective)-Center that referentially denotes the psychological perspective in which the sentence is situated.

## Introduction

### Theoretical Discussions on the Chinese Reflexive

**Figure 1:**
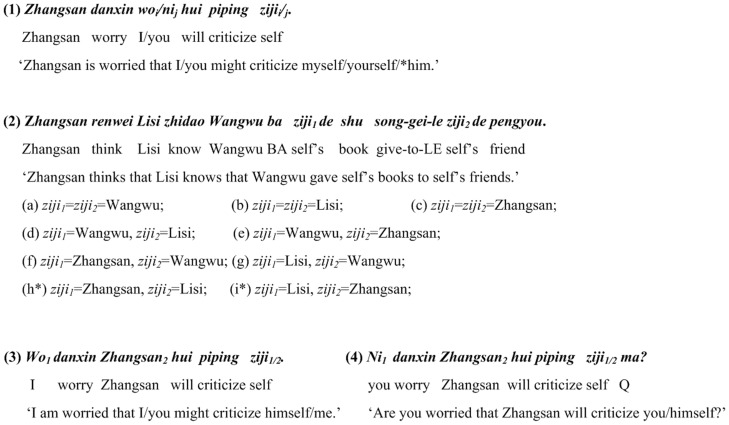
Example sentences showing the blocking effect on *ziji*. Sentence (1) has one occurrence of *ziji*. Sentence (2) has two occurrences of *ziji* (from [16], pp. 340 (36)). Sentences (3) and (4) show the exceptions to such blocking effect (from [2]). For each sentence, the first line shows the Roman spelling of this sentence, the second line shows the word gloss, and the third line shows the English translation. (a)–(g) are possible interpretations of the two *ziji*s in sentence (2). (h) and (i) are unacceptable ones, thus marked by “*”.

As is well discussed in the theoretical linguistic literature, there is a linguistic puzzle in Mandarin Chinese, the notorious reflexive *ziji* ‘self’ can take an antecedent across a clausal boundary, which contradicts Principle A of the Standard Binding Theory [1]. Meanwhile, *ziji* is subject to *a blocking effect*, i.e. a local 1st/2nd-person noun phrase (NP) may block a remote NP from being a long-distance antecedent, as illustrated by sentence (1) in Figure 1. Note that some scholar also pointed out that a local 3rd-person NP does not fully block a remote 1st/2nd-person NP from being a long-distance antecedent [2], as shown in sentences (3) and (4) in Figure 1.

Over the past three decades, many attempts have been made from different perspectives to characterize the distributional as well as referential properties of the Chinese reflexive. Despite many issues under considerable debate, there appears to be a general consensus that: (i) *ziji*, albeit notoriously uncharacterized, is subject to syntactic binding [3]–[5]; and (ii) its behavior is not purely syntactic, because semantic (e.g. [2], [6]–[8]) and pragmatic factors (e.g. [9]–[13]) also play significant roles. In other words, *ziji* has an ambiguous status in that it allows interpretation via either syntactic binding or discourse coreference [14], [15]. To be specific, there are two options for determining its antecedent. One is syntactic in nature: its referent is syntactically bound. The other is nonsyntactic in nature: its referent is “determined by nonsyntactic factors (semantic, pragmatic, discourse, processing, *inter alia*) whose nature remains largely obscure” ([14], p. 289).

Recently, some theoretical work has added more spice to the story of the notorious reflexive. For example, J. Huang, A. Li and Y. Li (henceforth HLL) [16], based on [17], went as far as to claim that even a 3rd-person NP was able to induce blocking for *ziji*. They constructed sentence (2) in Figure 1 and claimed that multiple occurrences of *ziji* could have mixed readings (e.g. (d)–(g) in Figure 1), as well as usual readings (e.g. (a)–(c) in Figure 1). HLL explained, as quoted: “The two occurrences of *ziji* may refer to the same antecedent, in which case any of the c-commanding subjects can be the antecedent (a, b, c). The two occurrences of *ziji* may also refer separately, so long as one of them is locally bound by *Wangwu* (d–g). Crucially, if both occurrences of *ziji* are to be LD [long-distance] bound, they must then be bound by the same long-distance antecedent (as in (b, c)), but not separately bound (as in (h, i)). This range of possibilities indicates that a 3rd-person NP does not induce blocking when it is itself a non-binder or local binder of *ziji*, but does so when it is itself a LD binder of *ziji*. In the illicit cases (h, i), the intermediate subject *Lisi* is the LD binder of one occurrence of *ziji*, and it prevents the other *ziji* from being bound by the matrix subject *Zhangsan*.” ([16], p. 341).

We feel that the encoding (not to mention the decoding) of a variety of coreference relationships between multiple *ziji*s and distinct antecedents as in HLL’s example is both conceptually and pragmatically implausible. With regard to the purported mixed readings ((d)–(g) in Figure 1) about multiple occurrences of *ziji*s, some crucial questions arise naturally from a conceptual perspective. For example, what are the semantic or conceptual mechanism underlying speakers’ encoding of, and the listeners’ decoding of, *a variety of* coreference relationships between multiple *zijis* and distinct antecedents? If Chinese syntax allows speakers to do so, it should also allow listeners to un-problematically decode these seemingly *chaotic* coreference relationships in a single sentence. Unfortunately, HLL did not offer any explanation of the mechanisms involved in encoding (and decoding) of those multiple coreference relationships. Pragmatically, it also seems rather difficult to accept HLL’s claim. *Ziji*, if occurring more than once in a single sentence, must be bound by one and the same antecedent for interpretation. The reason is simple: when expressing a sentence, a speaker can and must select only one Perspective-Center (in analogy to the deictic center) which referentially denotes the psychological perspective of the speaker from which the sentence is situated [8].

**Figure 2:**
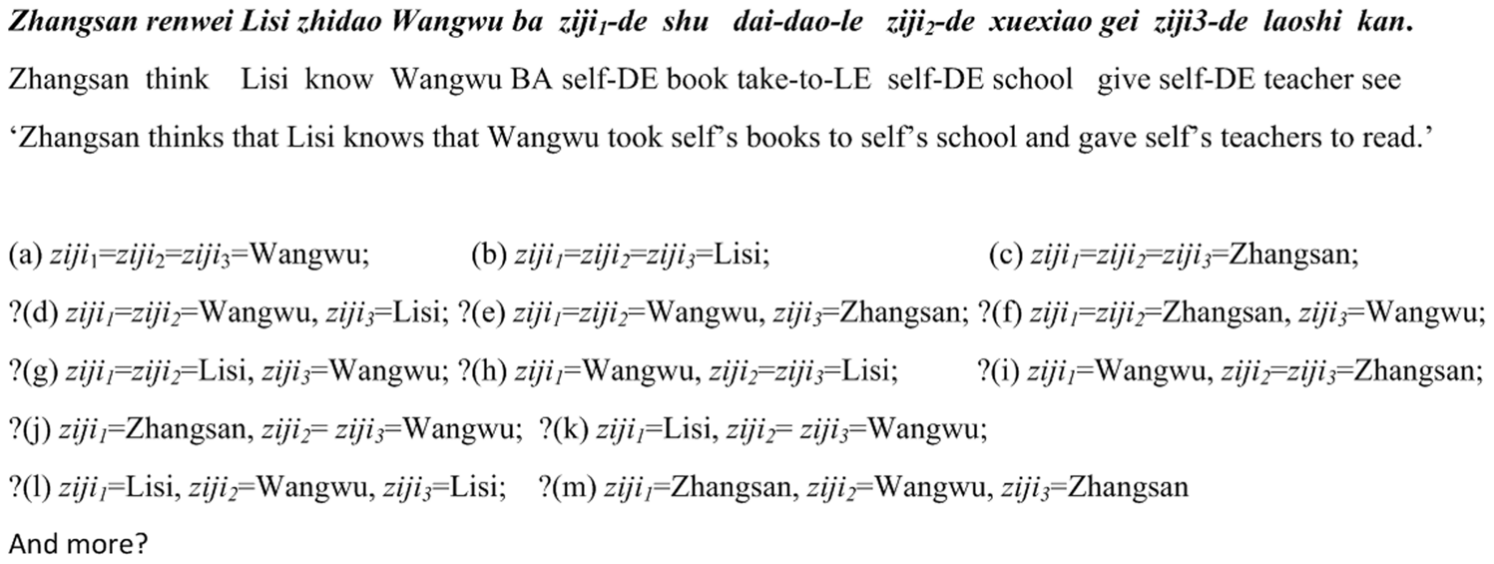
An example sentence having three occurrences of *ziji*. (a)–(m) are possible interpretations of the three *ziji*s. “?” indicate those interpretations might not be widely accepted by native Mandarin speakers.

And more importantly, if HLL’s syntactic characterization of multiple occurrences of *ziji* is on the right track, we should accordingly be able to predict the outcomes of interpreting sentences like the one in Figure 2, in which there are three *ziji*s. If this sentence is amenable to a treatment like HLL’s, we might follow their analysis and predict that in addition to the three possible readings in which all *ziji*s just take the same antecedent ((a)–(c) in Figure 2), there could be a great variety of possible outcomes of comprehending the sentence, including those mixed readings (e.g. (d)–(m) in Figure 2).

Undoubtedly, it is impossible to undertake such a referent-identification task. The implausibility of encoding a variety of coreference relationships between multiple *ziji*s and distinct antecedents in a single sentence will be seen more clearly, if we add more potential long-distance antecedents (note that the conceptual insolubility always exists if two or more *zijis* are purported to take distinct antecedents) to the sentence in Figure 2, e.g. *Niangzi shuo Mazi xiangxin Zhangsan renwei*… (‘lit. Niangzi say Mazi believe Zhangsan think …’), it will naturally create more different coreference relationships.

### Processing Experiments on the Chinese Reflexive

Apart from theoretical discussions, there are several experimental investigations of how the Chinese reflexive is processed in real-time. For example, Gao and colleagues conducted a cross-modal priming experiment asking participants to disambiguate *ziji*’s reference in sentences without any discourse context (e.g. *Laoshi gaosu jizhe yao zunzhong ziji* ‘The teacher told the newsman to respect himself’) [18], and found that the NP closest to *ziji* (i.e. the newsman) would be taken as the possible antecedent, thus echoing the effect of local binding as formulated by Principle A. They also conducted another experiment asking participants to disambiguate the reference of the Chinese pronoun *ta* ‘him’ in sentences without any discourse context, and found that unlike the reflexive, the pronoun’s resolution was not constrained by the governing category, which was also in agreement with Principle B of the Standard Binding Theory [1]. Using the same design and critical stimuli in [18], but variable stimulus onset asynchrony between *ziji* and the target, Liu conducted a lexical decision experiment [19], and discovered that the local binding between *ziji* and the local subject dominated over the long-distance binding between *ziji* and the long-distance subject, although the latter could took over in a later stage of processing.

Apart from these behavioral experiments, Li and Zhou conducted an ERP study and reported that the selection of a matrix subject as the long-distance antecedent of *ziji*, which was a violation of Principle A, engendered processing demands and hence incurred processing costs during online sentence comprehension [20]. In another ERP study, Schumacher and colleagues investigated how *ziji* was processed in sentences containing different types of verbs (i.e. self-oriented and distant-oriented) and different features of intervening NPs (e.g. 1st- and 2nd-person pronouns blocking *ziji*’s dependencies with more distant 3rd-person antecedents) [13]. Based on their ERP data, they presented a speech act account that offered support for the influence of both verb semantics and blocking effects during the comprehension of *ziji*.

All these experimental studies, basically in line with some theoretical discussions (e.g. [14], [15]), have provided some empirical evidence about the nature of the Chinese reflexive: (i) its resolution is syntactic in the sense that local binding has a higher priority in resolving its reference; (ii) if the long-distance binding occurs, it incurs processing costs, which may be due to semantic or pragmatic factors; (iii) both verb semantics and perspective-oriented pragmatics affect *ziji*’s resolution, with the blocking effect emerging as a later effect. In a word, there appears to exist an *antecedent-determining hierarchy* concerning *ziji*’s resolution: in general, its antecedent is determined via syntactic binding, which may be overridden by verb semantics, which may in turn be overridden by perspective-oriented pragmatics.

Now, the remaining question is: *What could happen when two occurrences of ziji in a single sentence are processed in real-time?* In this paper, we present an experimental investigation on how two occurrences of *ziji* are accessed and whether or not the processing of two *ziji*s is basically the same as that of one *ziji*, with the purpose of offering a new kind of evidence that contributes to the theoretical discussion. We focus on whether or not the referents of two *ziji*s in a single sentence can be determined in a principled way as claimed by HLL, viz. whether or not both *ziji*s in a same sentence have the same antecedent(s), and if mixed readings take place, whether or not they are patterned, albeit chaotic on the surface, out of the so-called blocking effect induced by a 3rd-person NP.

## Methods and Results

We conducted two sentence reading experiments following a modified self-paced reading paradigm. In these experiments, the self-paced reading introduced an online interpretation environment. However, unlike previous self-reading experiments that focused on the reaction times used by participants to read individual words in test sentences (e.g. [21]–[23]), we concentrated on the patterns of associating *ziji* with antecedents indicated by the answers to predefined questions toward test sentences and the reaction times used by participants to answer these questions. This information revealed explicitly how participants resolved the two *ziji*s in these complicated sentences, yet such interpretation patterns could not be clearly detected based simply on the reading times of individual words. Another reason for such modification was to avoid the possible effects caused by participants’ short-term memory during online reading of sentences involving deeply-embedded structures, because the standard self-paced reading paradigm was primarily used to examine real-time processing of general, spoken sentences, whereas the test sentences in our experiments (similar to sentence (2) in Figure 1) were much more complicated and less frequent in daily conversation.

Participants in these experiments perform slightly different reading tasks. In Experiment 1, participants were instructed to provide a prompt answer to test questions so that other factors, such as those caused by long-time thinking, could be avoided. In order to evaluate the effects of memory, in Experiment 2, participants were allowed to see test sentences while answering questions. Given that the test sentences in our experiments have been well discussed in theoretical linguistic literature but have never been dealt with in psychological experiments, our two experiments are a kind of compromise between linguistic paper-and-pencil tests and psycholinguistic sentence processing experiments. Although these two experiments have their own limitations in the sense that their procedures are not wholly online, Experiment 2 in particular, they are novel in the sense that they target on participants’ interpretation of multiple *ziji* in an online reading environment and take into account the effect of memory during online reading.

During these experiments, we are concerned primarily with two questions: (i) whether or not two *ziji*s co-existing in a same sentence should have the same references; and (ii) whether or not two *ziji*s could have mixed interpretations (i.e. they are interpreted as being bound by distinct antecedents), and if mixed interpretations would happen, whether or not they are patterned as claimed by HLL (construed as in a principled way). If mixed readings would indeed pattern like (d)–(g) in Figure 1, the blocking effect induced by a 3rd-person NP, as claimed by HLL, can be confirmed. If mixed readings would not pattern in a principled way as claimed by HLL, i.e. they would be essentially chaotic, then, the so-called blocking effect can be disconfirmed, and this would also support to a great extent our prediction that Chinese syntax should not allow multiple occurrences of *ziji* in a same sentence to take distinct antecedents, i.e. such cases are illicit in Chinese syntax, though we need to account for why there exist chaotic readings at all. Given that, at least mathematically, there are a great variety of possibilities of assigning referents to two or three *ziji*s in sentences like those in Figures 1 and 2, such as the local subject NP, the intermediate subject NP and the matrix or topmost subject NP, we would not unrealistically expect all participants to associate both *ziji*s with one and the same antecedent, viz. we would expect mixed readings to arise as a part of our results.

### Experiment 1

#### Purposes

This experiment investigated: (i) how Mandarin speakers determine the antecedents for the two occurrences of ziji in a same sentence like sentence (2) in Figure 1 without any discourse context during online-like sentence comprehension, and (ii) whether or not their referent identification is largely subject to syntactic binding as confirmed in previous experimental studies. It was approved by the College Research Ethics Committee (CREC) of the University of Hong Kong.

#### Participants

Thirty-five native Mandarin speakers (23 females and 12 males, among whom 20 came from North China and 15 South China) from the University of Hong Kong, aging from 20 to 32 years old (mean: 25.9±2.8), volunteered to participate in this experiment. All of them had normal or corrected eyesight, and no history of head trauma according to self-report. All of them signed consent forms before the experiment and were paid 80 HKD for participation after the experiment.

#### Materials

**Figure 3:**
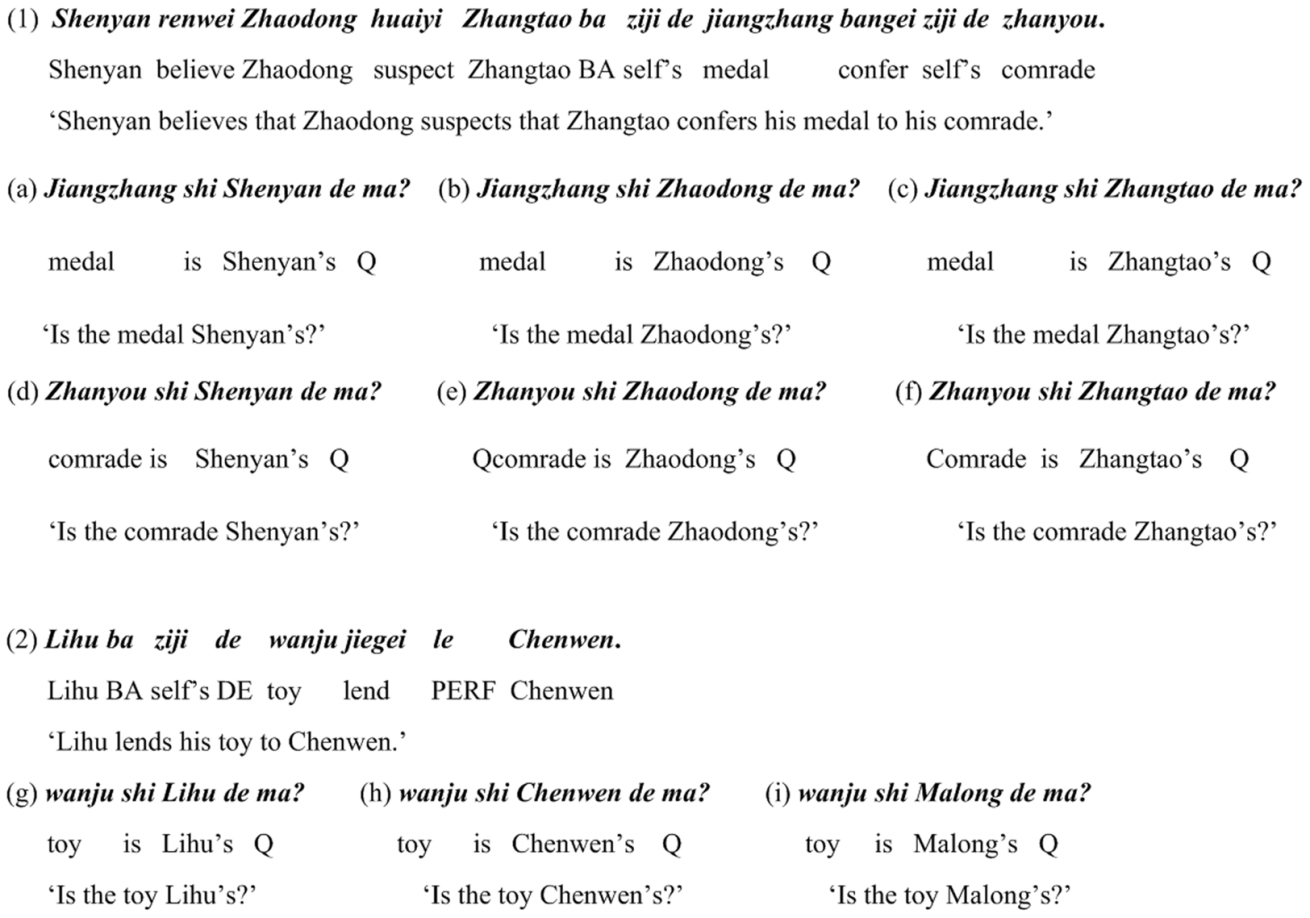
Examples of a test sentence (1) with its 6 test questions (a)–(f) and a filler sentence with its 3 questions (g)–(i). For each sentence or question, the first line shows the Roman spelling of this sentence, the second line shows the word gloss, and the third line shows the English translation.

We constructed 15 test sentences in this experiment (see Figure S1 for the list of all test sentences), each having 2 occurrences of *ziji*. For each sentence, we designed 6 test questions to detect how the two *ziji*s were associated with the three potential antecedents in the sentence (i.e. the three subjects in the main and embedded clauses of the sentence, which were numbered as the 1st, 2nd, and 3rd subject, among which the 3rd subject is the local antecedent). Figure 3 shows an example of the test sentence (sentence (1)) and its 6 test questions (a)–(f). Apart from test sentences, we also constructed 2 filler sentences, each having one occurrence of *ziji*. For each filler sentence, we designed 3 questions to test whether or not participants could correctly resolve the unambiguous antecedent of *ziji*. The purpose of inserting such filler sentences was to detect whether or not participants were actively engaged in the task. Figure 3 also shows an example of the filler sentence (sentence (2)) and its 3 questions (g)–(i). Note that the blanks within a test or filler sentence were used to split the whole sentence into individual words, which appeared one by one when participants pressed the SPACE bar during the self-paced reading. In the actual experiment, the sentences presented on the screen did not contain such blanks.

The reason why we asked about the referents of *ziji*s separately instead of asking about them simultaneously was to encourage participants to treat each occurrence of *ziji* individually and avoid the interferences between multiple occurrences of *ziji* in resolving all the possible anaphoric relationships. In order to test whether there is a consistent result, we presented the questions of each sentence to the participants for multiple times. Since participants were asked to answer each question based on their judgments after seeing each sentence, and they were allowed to have inconsistent understanding of the same sentence they had already seen, there was a chance to associate or not associate a *ziji* with a specific antecedent. Moreover, the order of all questions was randomized across blocks for each participant, so that the influences from previously presented sentences and questions relating to *ziji* in the current question were neutralized.

#### Procedure

The experiment was conducted in a quiet room. Participants sat in front of a computer, the distance between their eyes and the computer screen was 80 cm, and the horizontal vision angle was 4 degree. During the experiment, they were instructed to read sentences displayed on the screen in a self-paced fashion, and answer questions about those sentences immediately after reading them. In each trial, a fixation point initially appeared in the center of the screen for 500 ms. Then, the participant performed a self-paced reading, by pressing the SPACE bar on the keyboard with his/her left hand. Along with the SPACE bar pressing, one of the test or filler sentences gradually appeared on the screen word-by-word, from left to right, and ending with a period. The color of the background screen was black. The font of the displayed Chinese words was Simsun, the font size was 60, and the color was white. After the whole sentence was presented, the participant could press the SPACE bar again, and one of the six questions for that test sentence or one of the 4 questions for that filler sentence appeared in the middle of the screen, replacing the test or filler sentence. The participant had to answer this question by pressing the left or right mouse button with his/her right index or middle finger. The left or right button was tagged ‘Yes’ or ‘No’, and the tagging was randomized across participants. The whole experiment was designed and implemented using the E-Prime software (ver. 1.1).

The experiment started with 3 practice trails for participants to familiarize themselves with the experiment, and then, 3 sessions of experimental trials. There were in total 270 (15 sentences×6 questions×3 repetitions) test trials and 36 filler trials, evenly distributed in 3 sessions each containing 6 blocks. Each block contained a random sequence of the 15 test sentences together with one of the 6 test questions, plus the 2 filler sentence with one of the 3 questions randomly inserted among the 15 sentences. In each session, all test questions for all test sentences were shown only once. The presentation sequence of the test sentences and the display of one of the 6 questions after each test sentence were randomized in each session and across participants. Participants were allowed to take a 1-minute rest after each block and a 5-minue rest after each session. The whole experiment lasted around 1.5 hours.

#### Results

All participants had over 97.2% correctness in answering questions to the filler sentences. We exported all participants’ ‘Yes’/‘No’ responses to the test questions from the E-Prime for analysis. We first evaluated the reliability of participants’ responses to the test questions. The Cronbach’s *α* across the three repetitions was above 0.8 for all participants, indicating that each participant provided largely consistent answers to each test question across the three repetitions. Then, we analyzed these responses. Since these test questions were independent and only one of them appeared after each round of the presence of the test sentence, we can simply detect the association of a *ziji* with an antecedent by examining the responses to each of the six questions about a particular test sentence. The response to a certain question was regarded as ‘Yes’ (denoted by 1), if the participant responded ‘Yes’ to this question for at least two times out of the three repetitions; otherwise, the response was regarded as ‘No’ (denoted by 0). Apart from the responses, we also analyzed the reaction times to the test questions. The reaction time data were normalized in a log scale, so that they followed normal distributions. Outliers that exceeded 2.5 times of the standard deviation away from the mean reaction time were discarded.

After quantifying and normalizing the data, we input the response and reaction time data to the SPSS software (ver. 18.0) for statistical analysis. We conducted a two-way repeated-measures ANOVA test. There were two within-subject factors: *reflexive*, whose two levels corresponded respectively to the 1st and 2nd *ziji* in each sentence; and *antecedent*, whose three levels corresponded respectively to the three subjects in the main and embedded clauses with which the two *ziji*s were possibly associated.

As for the response data, the ANOVA test showed that the antecedent factor had a significant main effect on the responses (*F*(1.576, 53.578) = 46.261, *p*<.0005, *η^2^* = 0.500; *η^2^* was calculated based on the sum of squares, apart from *η_p_^2^* shown in SPSS). This indicated that distinct antecedents had significantly different degrees of being linked with the two *ziji*s. The ANOVA test also showed that the reflexive factor did not have a significant main effect (*F*(1, 34) = 2.957; *p* = 0.095, *η^2^* = 0.0002). This revealed that the participants were less likely to link both *ziji*s with two distinct antecedents. Moreover, the ANOVA test revealed a marginally significant interaction between the reflexive and antecedent factors (*F*(2, 68) = 3.022, *p* = 0.055, *η^2^* = 0.011). This showed that there was a similar trend in associating the three subjects with the two *ziji*s. All these suggested that the participants tended to consistently associate both *zijis* with the same antecedent(s).

**Figure 4:**
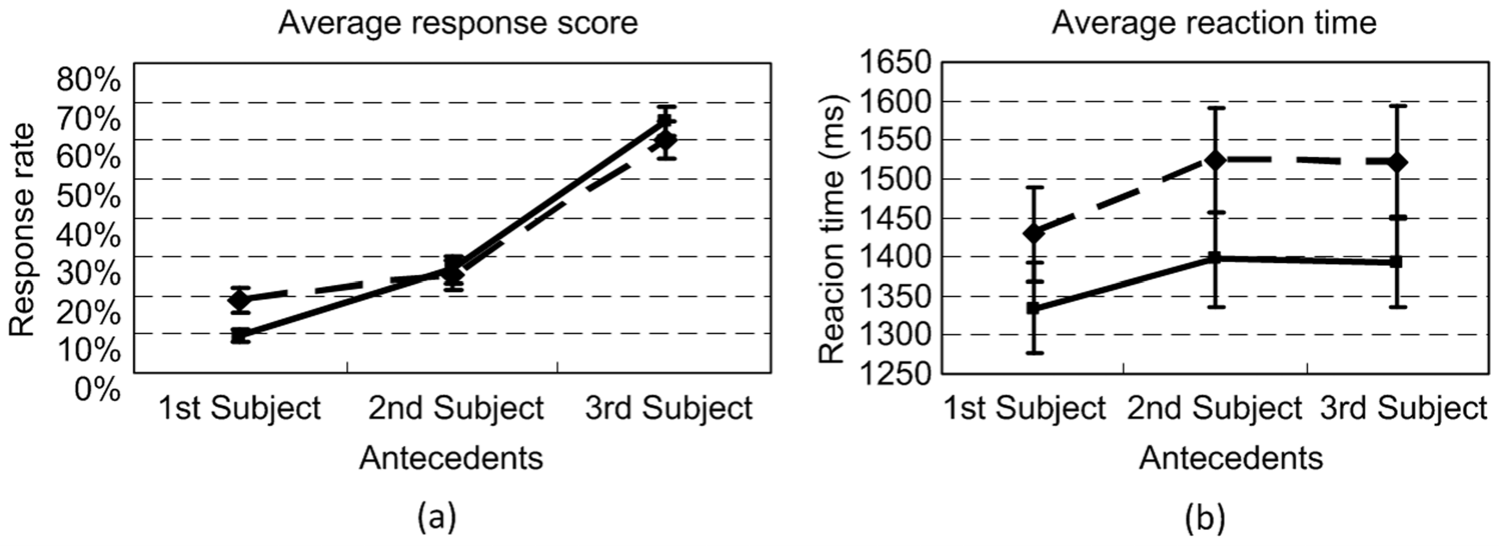
Results of Experiment 1: The average response scores (a) and reaction times (b) of the 6 ways of resolving the two *ziji*s by all participants. The solid line with diamonds denotes the scores and the reaction times of the 1st *ziji* in the test sentence, and the dashed line with blocks denotes the scores and the reaction times of the 2nd *ziji* in the test sentence. Each error bar indicates one standard error.

Figure 4(a) shows the average response scores and their standard errors across all participants. It is shown that the general trend was to associate both *zijis* with the 3rd subject (the local antecedent). Such trend took up over 60% of all responses. A post-hoc T-test confirmed that the chance of associating both *zijis* with the 3rd subject was significantly higher than that of associating them with the 1st (*p*<.0005) or 2nd (*p*<.0005) subject. Similar differences also existed in the responses to the 1st and 2nd subjects (*p*<.003). Nonetheless, there were ten participants who occasionally identified the reference of the two *zijis* with distinct antecedents in some sentences, unlike the general and consistent pattern shown in mots participants’ responses.

Apart from the responses to all test sentences, we also examined the responses to each test sentence. As regards a test sentence, if participants tended to associate both *zijis* with distinct antecedents, the reflexive and antecedent factors should show a significant (or marginally significant) interaction. Among the 15 test sentences (see Figure S1), such significant interaction did occur in sentences (1) (*p*<.005), (6) (*p*<.010), (9) (*p*<.0005), (10) (*p*<.011), (11) (*p*<.002), (12) (*p*<.013) and (14) (*p*<.0005). When perceiving some of these sentences, those 10 inconsistent participants associated the two *zijis* with two distinct antecedents. We will discuss these inconsistent participants in the next section.

As for the reaction time data, a similar ANOVA test revealed significant main effects of both the reflexive (*F*(1, 34) = 38.868, *p*<.0005, *η^2^* = .201) and antecedent (*F*(2, 68) = 7.673, *p*<.001, *η^2^* = .078) factors, but no significant interaction between the two (*F*(2, 68) = 0.575, *p* = 0.565, *η^2^* = 0.003). Figure 4(b) shows the average reaction times to the test questions. It is shown that the participants spent significantly longer time judging which antecedent the second *ziji* was associated with, compared to the first *ziji*. They also needed relatively longer time to resolve the coreference relation between one *ziji* and the 2nd or 3rd subject than between one *ziji* and the 1st subject (*p*<.0006 and *p*<.0005 respectively), but there was no significant difference between the reaction times with regard to the 2nd and 3rd subject (*p* = 1.000). These indicated that the participants spent extra time determining the antecedent of the second *ziji* with respect to the 2nd or 3rd subject. Admittedly, this could be due to the participants’ short-term memory, which was avoided in Experiment 2.

### Experiment 2

#### Purposes

This experiment investigated how Mandarin speakers resolve the references of two *ziji*s in test sentences when they have chance to re-analyze these sentences, comparable to the offline paper-and-pencil tests. It was also approved by the CREC of the University of Hong Kong.

#### Participants

Twenty native speakers of Mandarin (9 females and 11 males, among whom half came from North China and half came from South China) from the University of Hong Kong, aging from 18 to 35 years old (mean: 25.9±4.0), volunteered to participate in this experiment. None of them took part in Experiment 1. All of them had normal or corrected eyesight, and no history of head trauma according to self-report. All of them signed consent forms before the experiment and were paid 60 HKD for participation after the experiment.

#### Materials

**Figure 5:**
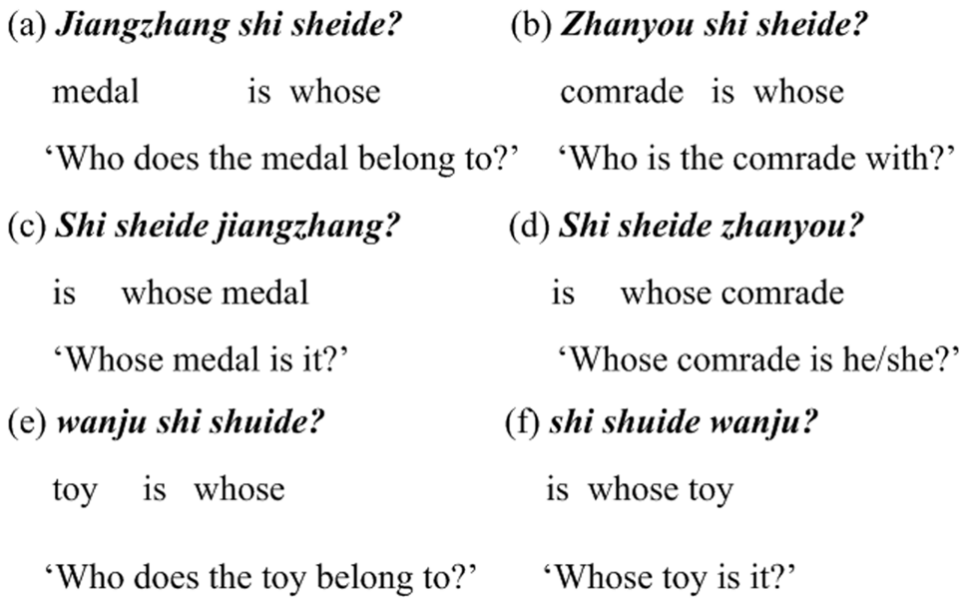
Test questions ((a)–(d)) for the test sentence (1) and questions ((e) and (f)) for the filler sentence (2) in Figure 3 in Experiment 2. Questions (a), (b), and (e) have one type of voice, and (c), (d), and (f) have another type of voice.

This experiment used the same test and filler sentences as in Experiment 1. Due to the different types of answers, the test questions became different. Unlike the ‘Yes’/‘No’ questions in Experiment 1, we designed 4 types of test questions asking participants to explicitly point out one of the potential antecedents in test sentences. Similarly, we designed 2 types of questions asking participants to clarify the unambiguous antecedent in filler sentences. Figure 5 shows the 4 questions ((a)–(d)) about the same test sentence and the 2 questions ((e) and (f)) about the same filler sentence as in Figure 3.

#### Procedure

This experiment was conducted in the same environment as Experiment 1. In each trial, the modified self-paced reading procedure was identical to that used in Experiment 1. The only difference lied in the question answering part. In Experiment 1, the question replaced the test or filler sentence after the self-paced reading. In Experiment 2, the sentence would remain on the screen when the question appeared below it, so that the participants had sufficient time analyzing the sentence before answering the question. Both the question and the sentence disappeared, when participants pressed the key ‘1’, ‘2’, or ‘3’ on the keyboard to indicate which subject in the test sentence was the answer to the question.

Since there were three possible answers to each of the four questions about the test sentences, it needed more than three times of repetition of these questions to detect the consistent patterns in participants’ responses. Therefore, in Experiment 2, each of the four test questions was repeated four times. In total, there were 240 test trials (15 sentences×4 questions×4 repetitions) and 32 filler trials, evenly distributed in four sessions each containing four blocks. In each block, each of the 15 test sentence together with one of the four test questions was shown only once, and the two filler sentence with one of the two questions was randomly inserted among the 15 sentences. In each session, all test questions for all test sentences were shown only once. The presentation sequence of the test sentences and the display of one of the four questions after each test sentence were randomized in each session and across participants. Participants could take a one-minute rest after each block and a five-minute rest after each session. The whole experiment lasted around one hour.

#### Results

All participants had over 96.9% correctness in answering questions to the filler sentences. The Cronbach’s *α* across the four repetitions was above 0.8 for all participants, indicating that each participant provided largely consistent answers to each test question in the 4 sessions. Participants’ responses ‘1’, ‘2’, and ‘3’ to the four types of test questions were transformed to the ‘Yes’/‘No’ answers similar to Experiment 1. For example, if the response was ‘1’, it equaled to a ‘Yes’ answer when a *ziji* was associated with the 1st subject. After the transformation, the scores were converted to ‘1’ or ‘0’ using the same way as in Experiment 1. With regard to a particular question, if the majority of the responses were pointed to a particular antecedent, the score was ‘1’ for that antecedent. If no answer reached the majority in the total number of repetitions, the data were excluded. In total, only two responses were excluded at the screening stage. In addition, the reaction times to the test questions were also transformed to a log scale to match normal distributions. Outliers that exceeded 2.5 times of the standard deviation away from the mean reaction time were discarded.

As regards the response data, a three-way repeated-measures ANOVA was conducted. Unlike Experiment 1, this ANOVA test involved three within-subject factors: reflexive, antecedent, and *voice of the questions* (for example, in Figure 5, questions (a) and (b) were asked in one type of voice, and (c) and (d) another type of voice). As for the reaction times, a two-way repeated-measures ANOVA was conducted, with reflexive and voice of the questions taken as two within-subject factors.

**Figure 6:**
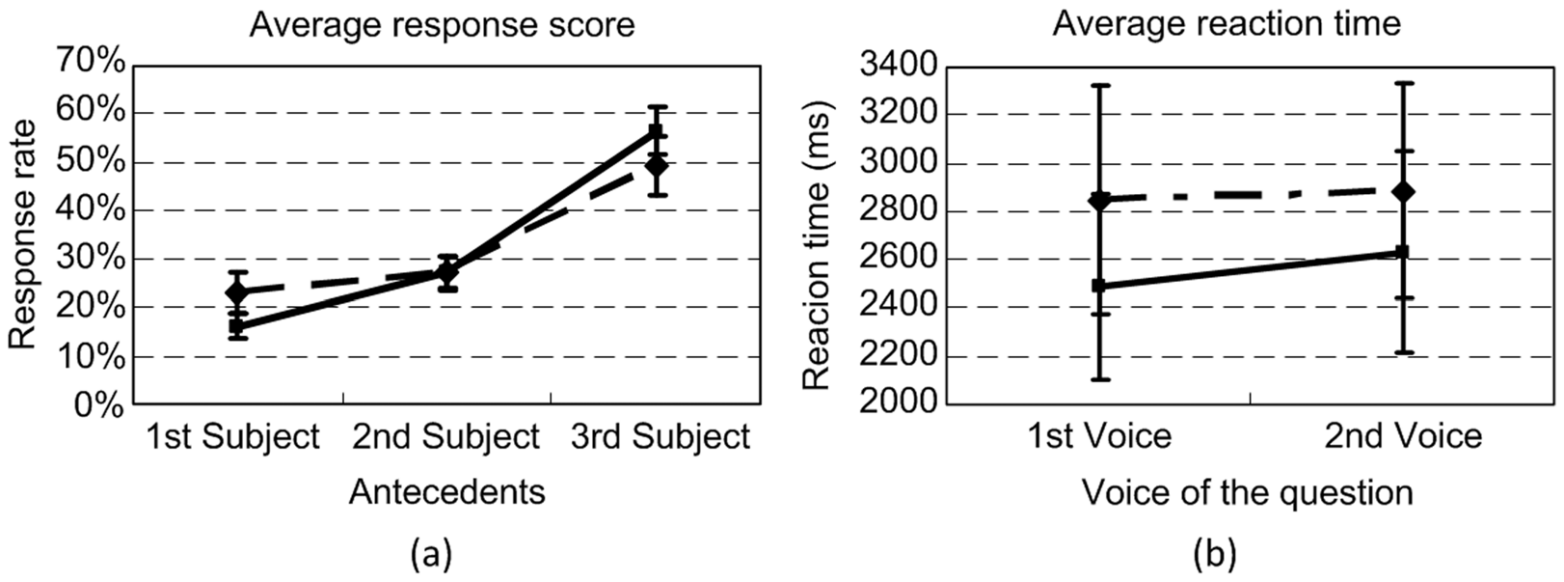
Results of Experiment 2: (a) Transformed average response scores of the 6 ways of resolving the two reflexives by all participants; (b) Averaged reaction times to the 4 types of questions. The solid line with diamonds denotes the scores and the reaction times of the 1st *ziji* in the test sentence, and the dashed line with blocks denotes the scores and the reaction times of the 2nd *ziji* in the test sentence. Each error bar indicates one standard error.

The ANOVA test revealed a significant main effect of the antecedent factor on the responses (*F*(1,363, 25.904) = 13.118, *p*<.0005, *η^2^* = .355), but no other significant main effects or interactions. The non-significance of the voice of the questions showed that the ways of asking the questions did not affect the judgments made by the participants. Figure 6(a) shows the average response scores. A post-hoc analysis confirmed that the chance of associating both *ziji*s with the 3rd subject was significantly higher than that of associating them with the 1st (*p*<.001) or 2nd (*p*<.012) subject. There was no significant difference between the responses to the 1st and 2nd subjects. Like Experiment 1, these results indicated that most participants tended to associate both *zijis* with the same antecedent, i.e. the 3rd subject in the test sentences. Nonetheless, there were four participants who occasionally associated the two *zijis* with distinct antecedents in some sentences, which was distinct from the general pattern.

We also examined the responses to every test sentence to see if there were significant (marginally significant) interactions between the reflexive and antecedent factors. Among the 15 test sentences (see Figure S1), such significant interaction occurred in sentences (1) (*p*<.036), (9) (*p*<.002), (10) (*p*<.027) and (14) (*p* = .087, marginal). When perceiving some of these sentences, those 4 inconsistent participants associated two *zijis* with two distinct antecedents. We will discuss these inconsistent participants in the next section.

As regards the reaction time data, a similar ANOVA test revealed a significant main effect of the reflexive factor (*F*(1, 19) = 17.815, *p*<.0005, *η^2^* = .176), but no other significant main effects or interactions. The non-significance of the voice of the questions showed that the ways of asking the questions did not affect the judgments made by the participants. Figure 6(b) shows the average reaction times. As in Experiment 1, a significant longer reaction time spent on the resolution of the second *ziji* than the first one was observed.

## Discussion

### General Pattern of Interpreting Multiple Occurrences of Ziji

Although Experiment 1 involved largely online processing, the results were basically consistent with those of Experiment 2. The correspondent results in both of these experiments clearly demonstrated a general way of resolving the reference of the two *ziji*s in the test sentences. More specifically, most native Mandarin speakers were consistent when determining the coreference relationships between the two *ziji*s and the three subjects as potential antecedents, even in two different judgment procedures: They tended to link both *ziji*s to the same antecedents, namely, the local subjects of the innermost clauses in which the two *ziji*s appeared. These findings suggest that the construal of multiple occurrences of *ziji* in a single sentence is largely subject to the Binding Principle A [1]. In other words, the referentially dependent reflexive, single or multiple, is syntactically bound by the local antecedent, when contextual information is not explicitly provided. This is compatible with previous experimental studies on a single occurrence of *ziji* (e.g. [18]–[20]).

Apart from this general interpretation pattern, there were some exceptions to the syntactically-based interpretation of the two occurrences of *ziji*. By comparing the responses to individual test sentences, we found that when perceiving the test sentences (1), (6), (9), (10), (11), (12), and (14) (see Figure S1), a small number (10 in Experiment 1 and 4 in Experiment 2) of the participants tended to associate the two *zijis* with two distinct antecedents. Most of these sentences involved some attitude verbs in the main or the first embedded clause. For example, in the test sentence (12) *Sunhong lijie Zhengwei taoyan Zhaopeng xiang ziji de pengyou toulou ziji de jihua* (‘Sunhong understands that Zhengwei dislikes that Zhaopeng tells his friends his plan’), the verb *taoyan* ‘dislike’ in the first embedded clause was an attitude verb with some emotional meaning. Consequently, a small number of the participants judged the 2nd (the immediate) subject *Zhengwei* as being the antecedent of the 2nd *ziji*. Similarly, in the test sentence (14) *Ligang haipa Wujia huaiyi Jinli ba ziji de cunkuan nuodao ziji de gongsi zhangshang* (‘Ligang fears that Wujia suspects that Jinli transfers his savings into his company’s account’), both the verb *haipa* ‘fear’ in the main clause and the verb *huaiyi* ‘suspect’ in the first embedded clause were attitude verbs with some emotional meaning. Then, a small number of the participants judged the 1st (the furthest) subject *Ligang* as being the antecedent of the 2nd *ziji*.

Nevertheless, attitude verbs alone were not responsible for such exceptions. For example, in the test sentence (13) *Qianyan yanwu Mengke yunxu Liting xiang ziji de shangci huibao ziji de tongshi* (‘Qianyan hates that Mengke permits Liting to report her colleague to her boss’), the attitude verb *yanwu* ‘hate’ did not prompt the participants to take an exceptional way of resolving the reference of the reflexives. Another example is the test sentence (6) *Liutao tingshuo Wangming qingqiu Zhangli ba ziji de chanpin mai gei ziji de duishou* (‘Liutao hears that Wangming asks Zhangli to sell his products to his opponent’). This sentence did not involve any attitude verb, but some participants still associated the two *zijis* with two distinct antecedents. This suggested that nonlinguistic factors could possibly prompt some participants to determine the reference of the two *zijis*, as will be discussed later.

To sum up, syntactic binding has the priority of determining the reading of *ziji*, single or multiple, albeit it may sometimes be overridden by verb semantics or perspective-oriented pragmatics.

### Mixed Readings and Blocking Effects

Apart from the largely consistent patterns, mixed readings of two *ziji*s (i.e. two *ziji*s were judged as having two distinct antecedents) did take place, but they did not appear to confirm HLL’s claim. In HLL’s claim, there is a principled way to account for mixed readings about the references of two *ziji*s, i.e. the mixed readings of two *ziji*s as indicated in (d)–(g) in Figure 1, albeit seemingly chaotic at first glance, are actually patterned as a display of veiled blocking effects which are induced by a 3rd-person NP. Our results showed that the so-called blocking effects did not emerge, since two *ziji*s were judged by the inconsistent participants as referring to the 2nd subject as well.

**Figure 7:**
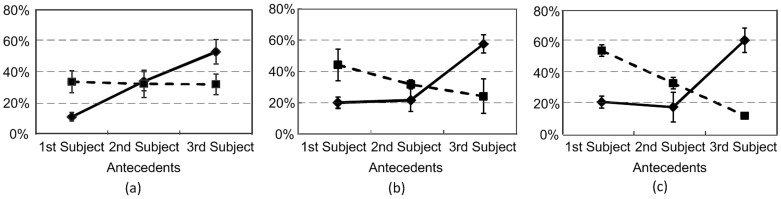
Response scores of inconsistent participants in Experiments 1 and 2: (a) Average response scores to the 6 ways of resolving the two reflexives by the 10 inconsistent participants in Experiment 1; (b) Transformed average response scores to the 6 ways of resolving the two reflexives by the 4 inconsistent participants in Experiment 2; (c) Transformed average response scores to the 6 ways of resolving the two reflexives by the 3 of the 4 inconsistent participants in Experiment 2. The solid line with diamonds denotes the scores and the reaction times of the 1st *ziji* in the test sentence, and the dashed line with blocks denotes the scores and the reaction times of the 2nd *ziji* in the test sentence. Each error bar indicates one standard error.

Figure 7(a) and Figure 7(b) respectively show the average response scores of the inconsistent participants in the two experiments (ten in Experiment 1 and four in Experiment 2). In addition, three out of the four inconsistent participants in Experiment 2 had rather similar response scores, but their scores were quite distinct from the other one, especially in associating the second *ziji*. Noting this, Figure 7(c) further shows the average response scores of these 3 inconsistent participants in Experiment 2. As shown in these figures, these inconsistent participants could select any of the three subjects as the potential antecedents for the two *ziji*s. Nonetheless, they tended to select the 3rd subject as the antecedent for the 1st *ziji* (53% among the 10 inconsistent speakers in Experiment 1 and 61% among the 4 inconsistent speakers in Experiment 2). This, to some degree, also demonstrated the effect of local binding. In addition, when checking the actual associations of these participants, we found that once the 2nd subject, namely a potential long-distance binder in HLL, was already chosen as one antecedent for one *ziji*, the 1st subject was also chosen as the other antecedent for the other *ziji*. This unambiguously invalidates HLL’s claim that a 3rd-person NP can induce blocking if it is itself a long-distance binder, because there should be no principle whatsoever to account for the mixed readings in our experiments.

As predicted at the outset, Chinese syntax would not possibly allow multiple occurrences of *ziji* in a same sentence to refer to distinct antecedents in all likelihood, because they are both conceptually and pragmatically impossible. The challenging question then is: *How can we account for the mixed readings which had no pattern at all?* In other words, given that mixed readings about multiple *ziji*s did occur in our experiments, *how can we say HLL’s claim that the multiple occurrences of ziji can take distinct antecedents would face some insoluble conceptual problems?* Since our data makes it hard to identify the underlying mechanisms (syntactic, semantic, pragmatic, or whatever works behind them), we have good reason to suspect that mixed interpretations occurring in our two experiments as well as offline judgments such as HLL’s appear to arise as a consequence of manifold, interlocking and conflicting perspectives.

Let us first address the role of perspective in the construction and comprehension of sentences involving the Chinese reflexive.

**Figure 8:**
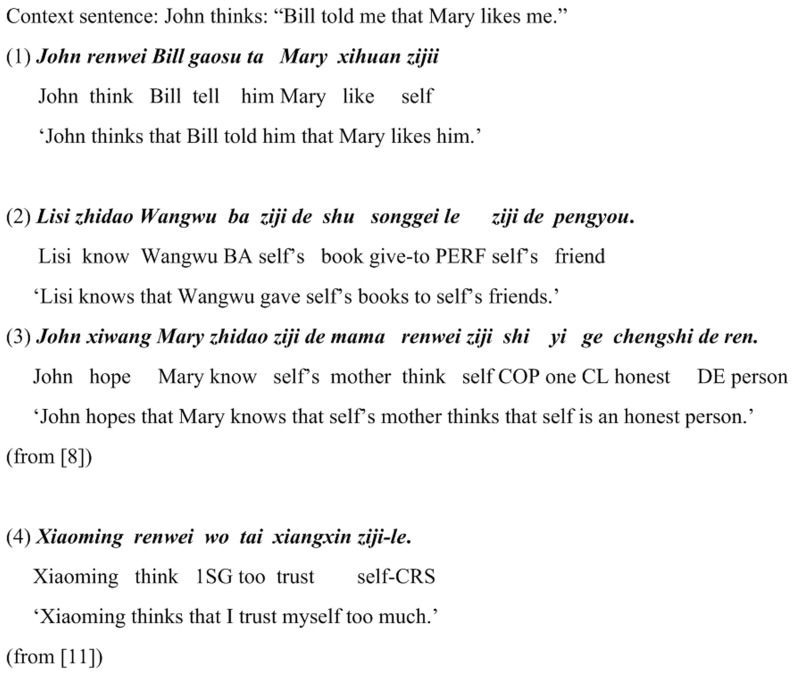
Test sentences used by previous studies: Sentences (1), (2), and (3) from [8], and (4) from [11].

In the antecedent-determining hierarchy of the Chinese reflexive (see Sec. 1), we explicitly state that although, in general, the antecedent of *ziji* is syntactically bound (i.e. local binding), syntactic binding can sometimes be overridden by perspective related to speaker- and listener- directed information, which gives rise to the so-called long-distance binding. In this regard, based on a range of long-distance binding tests, Anand offered a plausible account of how the semantic mechanism relating to perspective might work. In one of his tests, given a discourse-context (the context sentence in Figure 8), 29 Mandarin speakers were asked to judge the grammaticality of sentence (1) in Figure 8. The results showed that 16 of them considered this sentence ungrammatical and 13 grammatical. Based on a series of divergences in judgment among these speakers, he pointed out two different grammars used by these speakers for binding long-distance *ziji* in this sentence: (i) treating the reflexive as a Perspective-based shifting indexical, this grammar was used by those 13 speakers; and treating the reflexive as a discourse-dependent logophor, this grammar was used by those 16 speakers. As for the first grammar, Anand pointed out the two shifting indexicals (the pronoun and the *ziji*) must ‘work together’ to co-refer. Using sentences (2) and (3) in Figure 8, Anand went further to discuss multiple occurrences of *ziji* in a single sentence. According to him, the two *ziji*s must be bound by one and the same antecedent, which could be nicely accounted for by the two semantic properties (shifting indexical and discourse-dependent logophor). For example, if one *ziji* in (2) refers to the speaker, as pointed out by Anand, the other *ziji* must do as well, and cannot refer to *Lisi*. As with (3), if the second *ziji* is anteceded by *John*, *Mary* or the speaker, the other *ziji* must be as well. This is simply because, when expressing a sentence, the speaker can and must select only one P(erspective)-Center which is a point-of-view that “referentially denotes the psychological perspective from which the sentence is situated (in analog to the deictic center for a sentence)” ([8], p. 137). Thus, the semantic requirement that two or more *ziji*s must “shift together” naturally leads to the conclusion that long-distance Chinese reflexive binding is not syntactic but semantic in nature.

After pointing out that multiple *ziji*s in a single sentence must referentially co-refer due to the absolute constraint of P-Center, we now address the question of why mixed readings about multiple *ziji*s should exist at all.

With regard to the blocking effect induced by the 1st-person and 2nd-person pronouns as shown in sentence (1) in Figure 1, Y. Huang constructed the example as sentence (4) in Figure 8 and proposed a perspective-based account of it [11]: The blocking effect in this sentence is the consequence of conflicting perspectives of an internal speaker (*Xiaoming*) and an external speaker (*wo ‘I’*). Following many logophoricity-based accounts of long-distance reflexivization (e.g. [24]–[26], *inter alia*), Y. Huang proposed that the use of a long-distance reflexive in Chinese seems closely correlated with a logophoric point of view, roughly that of an internal protagonist as opposed to an external speaker [11]. With regard to the above Chinese sentence where the reflexive shows the blocking effect, Huang explained that logophoricity in Chinese can involve one and only one center of point of view (which is so-called *a 3rd-person point of view* in [24]), namely the relativized center of deixis, which may not be allowed to be switched. In sentence (4) in Figure 8, the intervening 1st-person pronoun *wo* ‘I’ introduces a new local of point of view, viz. a new external speaker, and clashes with the perspective center which is already introduced by *Xiaoming* in the subject position, and as a consequence, the latter wins and the blocking effect thus emerges.

This kind of perspective-based account shed light on the mixed interpretations concerning multiple occurrences of *ziji*. We can reasonably assume that mixed readings (i.e. two or more *ziji*s in a single sentence are judged by some speakers to have distinct antecedents) arise as a consequence of manifold, interlocking and conflicting perspectives. Precisely, in our experiments, the mixed readings (i.e. two *ziji*s’ linking with distinct antecedents shown in our experiments) were the results of conceptual confusion caused by some complex factors, such as the multiple possibilities of local- as well as long-distance binding of the Chinese reflexive with a single or multiple potential antecedents. This was confirmed by our participants’ informal post-test reports: The longer they thought about the test sentences, the more likely mixed readings were to take place.

## Conclusions

In this paper, we conducted two experiments on how multiple occurrences of the Chinese reflexive in a single sentence were processed, using identical materials and stimuli but different experimental procedures. The general interpretation pattern observed showed that in sentences with two *ziji*s, the referentially dependent reflexive was largely bound by the local subject when contextual information was not explicitly provided. This was compatible with previous experimental studies in the sense that, in general (i.e. without any specific discourse context), *ziji*’s reading is subject to syntactic binding.

In addition, the small proportion of the mixed reading results (linking two *ziji*s with distinct antecedents) did not pattern in a principled way as claimed in HLL. This demonstrated that a 3rd-person NP does not actually induce a blocking effect, whether it itself is a possible long-distance binder or not. A detailed analysis revealed that such mixed readings emerged as a consequence of manifold, interlocking and conflicting perspectives. Precisely, they were due to the conceptual confusion caused by some complex factors, such as multiple possibilities of local as well as long-distance binding of the Chinese reflexive with a single or multiple possible antecedents. We thus conclude that cases of multiple occurrences of *ziji* taking distinct antecedents are actually illicit in Chinese syntax, or probably the syntax of any other language, for the simple reason that the speaker, when expressing a sentence, can and must select only one P(erspective)-Center referentially denoting the psychological perspective from which the sentence is situated [8], [11].

Admittedly, due to the complex nature of sentences involving multiple *ziji*s, we adopted a compromised experimental paradigm between linguistic paper-and-pencil tests and psycholinguistic experiments of online sentence processing. In order to have a full picture of the syntactic as well as the semantic mechanism of producing and interpreting sentences involving multiple occurrences of the Chinese reflexive, we may need to improve the way of experiment on complex sentences, which paves the way for our future work.

## Supporting Information

Figure S1 **15 test sentences.** For each sentence, the first line is the Roman spelling of the Chinese sentence, the second line is the word gloss, and the third line is the English translation. In the actual experiments, there are no blanks between words in these sentences.
(TIF)
