# Supplementary figures and images for: Who Is Who? Interpretation of Multiple Occurrences of the Chinese Reflexive: Evidence from Real-Time Sentence Processing
Source: PLoS One. 2013 Sep 3;8(9):e73226. doi: 10.1371/journal.pone.0073226 (PMC3760907; doi:10.1371/journal.pone.0073226)

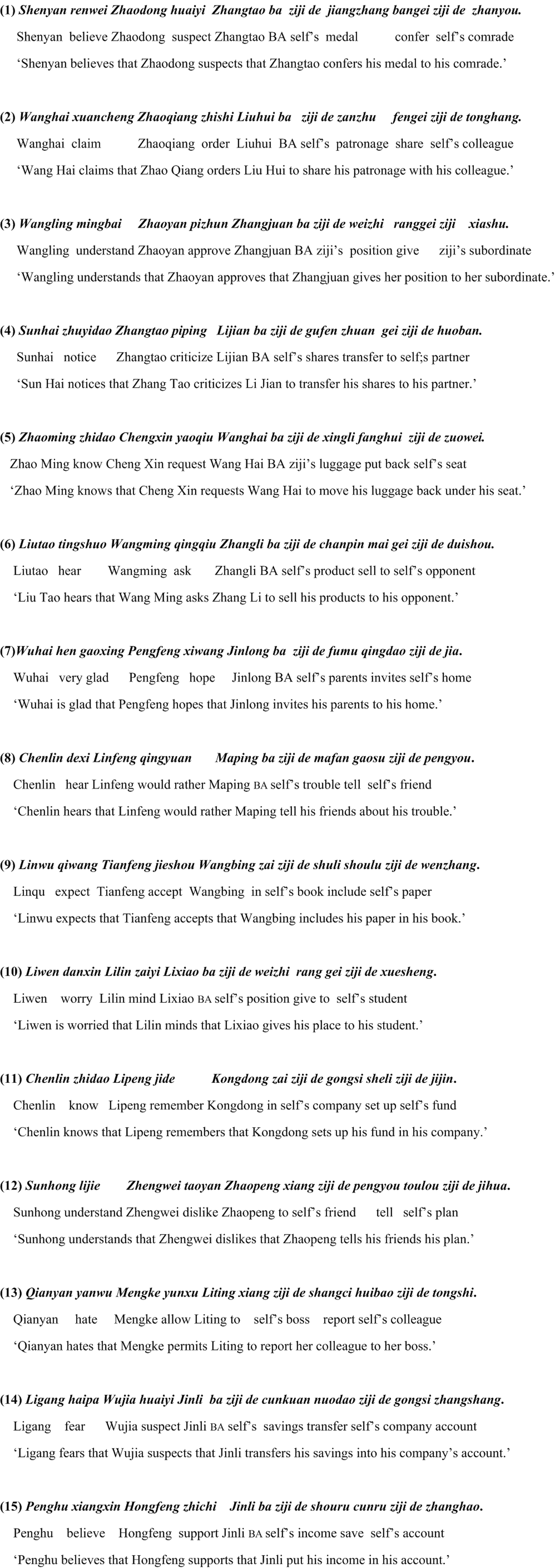

Supplement: Figure S1 — 15 test sentences. For each sentence, the first line is the Roman spelling of the Chinese sentence, the second line is the word gloss, and the third line is the English translation. In the actual experiments, there are no blanks between words in these sentences. (TIF) [file pone.0073226.s001.tif]
